# Influence of Nozzle Speed on the Crystallinity and Solubility of Polyvinyl Alcohol in Material Extrusion

**DOI:** 10.3390/polym17243320

**Published:** 2025-12-16

**Authors:** Ji Eun Lee, Yong Son, Seong Je Park

**Affiliations:** 1 Korea Additive Manufacturing Innovation Center (KAMIC), Korea Institute of Industrial Technology (KITECH), 113-58, Seohaean-ro, Siheung-si 15014, Gyeonggi-do, Republic of Korea; 2 School of Mechanical Engineering, Gyeongsang National University, 501 Jinju-daero, Jinju-si 52828, Gyeongsangnam-do, Republic of Korea

**Keywords:** additive manufacturing, material extrusion, polyvinyl alcohol, nozzle speed, dissolution rate

## Abstract

Material extrusion (MEX) commonly requires support structures, and their rapid removal is essential for improving overall process efficiency. This study investigates the effect of nozzle speed on the crystallinity and dissolution behavior of polyvinyl alcohol (PVA) supports fabricated by MEX. The measured crystallinity values are 28.8%, 25.4%, and 23.7% at nozzle speeds of 20 mm/s, 60 mm/s, and 100 mm/s, respectively. Dissolution rates are measured as 0.2144%/min, 0.2378%/min, and 0.2544%/min at nozzle speeds of 20 mm/s, 60 mm/s, and 100 mm/s, respectively. These results demonstrate that nozzle speed is a key parameter that governs both crystallinity and dissolution behavior of PVA. As a result, higher nozzle speeds not only shorten fabrication time but also produce supports with lower crystallinity. Thus, support structures can be removed more quickly and efficiently at high nozzle speeds. This work provides a new perspective on accelerating support dissolution, demonstrating that PVA crystallinity can be tailored through process parameter control in MEX from a chemical viewpoint, rather than relying on conventional physical approaches.

## 1. Introduction

Additive manufacturing (AM), according to ISO/ASTM 52900, can be classified into seven major categories [1]. Among these, MEX has gained significant popularity in both educational and household environments due to its ease of operation and relatively low cost [2]. Most research on MEX has focused on evaluating the mechanical properties of printed specimens or developing optimal process parameters to enhance part performance [3,4,5,6]. While these studies are undoubtedly important, another critical but often overlooked aspect of MEX lies in the removal of support structures.

**Figure 1:**
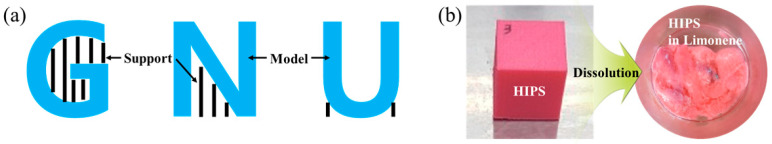
(**a**) Schematic of support and model. (**b**) Dissolution behavior of HIPS in limonene.

MEX processes inherently require the use of support structures, particularly when fabricating overhanging geometries to prevent collapse during the process, as shown in Figure 1a [7]. Support structures in MEX are generally categorized into soluble supports and breakaway supports [8]. Widely used commodity plastics, such as polylactic acid, acrylonitrile butadiene styrene, and polycarbonate, are often paired with soluble supports, which can be selectively dissolved in suitable solvents [9]. In contrast, super engineering plastics, such as polyetheretherketone and polyetherimide, are typically processed under high-temperature conditions, where the availability of compatible soluble support materials remains limited [10]. Among existing soluble materials, polyvinyl alcohol (PVA), which dissolves in water [11], and high-impact polystyrene, which dissolves in limonene as shown in Figure 1b, are the most widely used. PVA, in particular, has been widely adopted as a support material because of its excellent water solubility. The rapid removal of support is critically important in AM because prolonged dissolution times increase overall processing time. Therefore, developing strategies to accelerate support dissolution has significant implications for both manufacturing efficiency and part quality [12].

Numerous studies have shown that PVA dissolves faster at higher temperatures, in alkaline solutions, or with ultrasonication or stirring [13]. Further research has demonstrated that replacing water with hydrogen peroxide can also enhance dissolution rates [12]. Most of these studies have primarily focused on physical and environmental factors influencing dissolution. In contrast, there has been relatively little investigation into how process parameters during AM affect the crystallinity of PVA and, consequently, its dissolution behavior. This research gap limits the efficiency of post-processing in MEX, as slow support removal can significantly increase manufacturing time. Therefore, understanding how MEX process parameters influence the dissolution behavior of PVA is essential to improve both productivity and practicality of the MEX.

In this study, we address the lack of research on how AM process parameters influence the crystallinity of PVA and, consequently, its dissolution behavior. Our findings reveal that slower nozzle speeds lead to increased crystallinity, resulting in slower dissolution. In contrast, higher nozzle speeds not only reduce fabrication time but also produce PVA supports with lower crystallinity, which dissolve more readily. This demonstrates that process-driven crystallinity control offers a new practical strategy to enhance support removal in MEX.

## 2. Methodology

### 2.1. Materials

The filament-formed polyvinyl alcohol (PVA; ESUN, Shenzhen, China) with a 1.75 mm diameter was used to fabricate cubic specimens with dimensions of 10 × 10 × 10 mm^3^. Since PVA can absorb moisture from the environment, which may interfere with the dissolution test, the material was dried at 60 °C for 2 h before use. The material was selected because it is one of the most widely used water-soluble support materials for MEX processes.

### 2.2. Specimen Fabrication

Specimens were material-extruded (MEXed) using a custom-made MEX machine [14]. To ensure that the results reflect the typical characteristics of support structures, a cross-sectional area of 10 × 10 mm^2^ was selected. Support materials are generally intended to be removed after AM; therefore, they are typically fabricated with minimal volume, as shown in Figure 1a. Even when supporting large parts, supports are not printed as a full solid area but rather in sparse or discontinuous patterns. Hence, the chosen dimension reasonably represents the practical scale and geometry of support structures in MEX. The nozzle temperature, chamber temperature, bed temperature, fill density, layer thickness, and nozzle diameter were 200 °C, room temperature, room temperature, 100%, 0.2 mm, and 0.4 mm, respectively. The nozzle speed was controlled at 20 mm/s, 60 mm/s, and 100 mm/s.

### 2.3. Crystallinity Measurement

To measure the crystallinity of MEXed specimens according to the nozzle speed, the X-ray diffractometer (XRD; D8 Advance, Bruker, Billerica, MA, USA) was used. Diffraction patterns were obtained using Cu-Kα radiation (λ = 1.5406 Å) at 40 kV and 30 mA, with a scanning range of 2θ = 10–90° and a step size of 0.02°. The crystallinity was calculated as the ratio of the crystalline peak area to the total diffracted area, expressed as *X_c_* = *A_c_*/(*A_c_* + *A_a_*) × 100, where *A_c_* and *A_a_* represent the integrated areas of the crystalline and amorphous regions, respectively. All specimens (10 × 10 mm^2^) were tested after MEX without any post-annealing, ensuring identical thermal history and preparation conditions. All quantitative results of the dissolution test and XRD are displayed as the mean ± standard deviation based on four specimens per test.

### 2.4. Dissolution Test

The MEXed specimens were immersed in 500 mL of distilled water maintained at 80 °C in a beaker placed in a temperature-controlled bath. The dissolution test was conducted for 2 h under controlled conditions (80 °C in 500 mL of distilled water). After the dissolution test, the specimens were dried at room temperature for 24 h to calculate the dissolution rate. The dissolution rate of the MEXed specimen was calculated according to Equation (1):

Dissolution rate (%/min) = mass reduction rate (g/min)/initial mass (g) × 100(1)

where the mass reduction rate represents the decrease in mass per unit time, and the initial mass refers to the starting mass of the specimen. All quantitative results of the dissolution test are displayed as the mean ± standard deviation based on four specimens per test.

## 3. Results and Discussion

**Figure 2:**
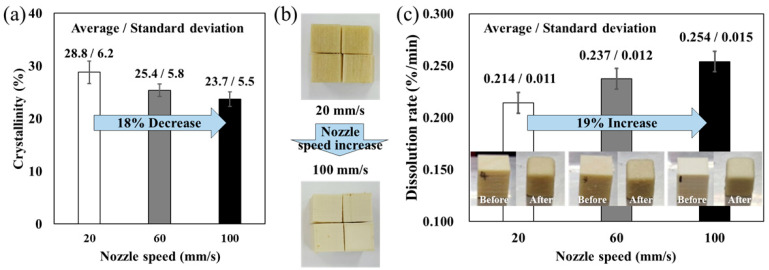
(**a**) Crystallinity, (**b**) color difference, and (**c**) dissolution rate of PVA according to the nozzle speed.

The crystallization of polymers generally occurs during cooling after extrusion. Slower cooling provides more time for molecular chains to arrange into ordered structures, thereby leading to a higher degree of crystallinity [15]. Previous studies have reported that higher nozzle speeds increase the average temperature of relatively large-area printed parts (19.1 × 31 mm^2^) [16]. Such elevated average temperatures within the specimen can promote crystallization. This explains why polymers MEXed under higher chamber temperatures generally exhibit higher degrees of crystallinity [14]. However, in this study, the specimens had a relatively small cross-sectional area of 10 × 10 mm^2^, making them much more sensitive to the localized heat input from the nozzle rather than bulk thermal accumulation. As the nozzle moved slowly across the small specimen area, it transferred heat for a longer duration, resulting in a higher average temperature of the part and providing sufficient time for crystal growth [17]. Consequently, the degree of crystallinity increased with decreasing nozzle speed. As shown in Figure 2a, the measured crystallinity values were 28.8%, 25.4%, and 23.7% at nozzle speeds of 20 mm/s, 60 mm/s, and 100 mm/s, respectively. Thus, the crystallinity decreased from 28.8% to 23.7%, corresponding to approximately a 17.7% reduction.

Previous studies on semicrystalline polymers such as PEEK have reported that a similar approximately 20% change in crystallinity can lead to noticeable physical and mechanical property variations in MEX-fabricated samples. Therefore, the observed 18% reduction in the present study is considered to be a meaningful structural change that can influence the dissolution behavior of PVA [12,15]. Furthermore, to verify the statistical significance of the observed trend, a one-way analysis of variance (ANOVA) was conducted based on the crystallinity data (*n* = 4 per condition). The result yielded F = 6.06 and *p* = 0.0215, indicating that the decrease in crystallinity with increasing nozzle speed is statistically significant at the 95% confidence level (The F-value represents the ratio of variance between the groups to the variance within the groups, and the *p*-value indicates the probability that the observed differences occurred by chance under the null hypothesis). A noticeable color difference was also observed among the MEXed specimens, as shown in Figure 2b. The samples printed at lower nozzle speeds exhibited a yellowish appearance, whereas those printed at higher speeds appeared beige. These color differences with crystalline differences are a well-known phenomenon in semicrystalline polymers [14]. This confirms that the observed variation is not due to random measurement fluctuations but represents a meaningful structural change in the MEXed PVA specimens.

**Figure 3:**
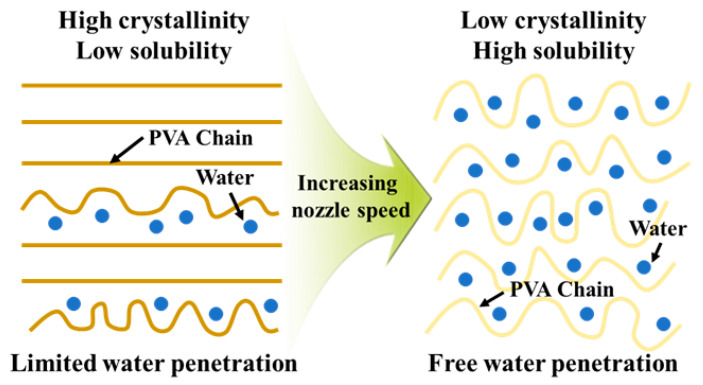
Schematic illustration for the effect of nozzle speed on crystallinity and dissolution behavior of MEXed PVA.

This structural evolution directly affected the dissolution behavior. Because higher crystallinity restricts solvent penetration and chain disentanglement, the dissolution rate decreased at slower nozzle speeds. Specifically, the dissolution rates were calculated as 0.214%/min, 0.237%/min, and 0.254%/min for nozzle speeds of 20 mm/s, 60 mm/s, and 100 mm/s, respectively, as shown in Figure 2c. Thus, increasing the nozzle speed fivefold (from 20 mm/s to 100 mm/s) enhanced the dissolution rate by approximately 19%. A one-way ANOVA was also performed on the dissolution rate (*n* = 4 per condition). The analysis yielded F = 4.17 and *p* = 0.052, suggesting that the increase in dissolution rate with nozzle speed is marginally significant at the 95% confidence level and becomes statistically significant when considering a 90% confidence level. These results clearly demonstrate that the specimens with higher crystallinity exhibited slower dissolution, whereas those with lower crystallinity dissolved more readily. Taken together, these findings indicate that nozzle speed acts as a critical process parameter governing both the crystallinity and dissolution behavior of PVA supports. The mechanism is schematically illustrated in Figure 3. Higher nozzle speeds not only reduce AM time but also yield supports with lower crystallinity, thereby enabling more efficient removal during post-processing.

**Figure 4:**
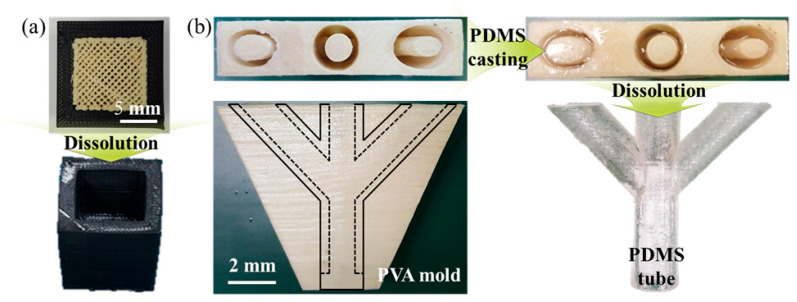
Application study to fabricate the (**a**) General PVA-removed model and (**b**) PDMS tube.

As illustrated in Figure 4, we fabricated a general model-PVA combination and PVA molds via MEX to further demonstrate the practical applicability of this fast dissolution-controlled strategy. After dissolution of the PVA mold, a well-defined general PVA-removed model and PDMS tubes were successfully obtained. This work introduces a new perspective for accelerating support dissolution by controlling process parameters in MEX to tailor PVA crystallinity in terms of chemistry, in contrast to conventional approaches focused mainly on a physical dissolution condition. Furthermore, the application study verified the feasibility of the proposed method in producing branched tubular structures.

## 4. Conclusions

This study confirms that nozzle speed can be an effective control parameter for tuning dissolution behavior of PVA supports fabricated by MEX. It was found that slower nozzle speeds increased crystallinity due to extended thermal exposure, which in turn reduced dissolution rates. Conversely, higher nozzle speeds resulted in lower crystallinity and faster dissolution. These findings have practical implications for optimizing post-processing in polymer AM, particularly for multi-material with soluble-support applications where fast and clean support removal is critical. This work introduces a new perspective for accelerating support dissolution by showing that PVA crystallinity can be tailored through process parameter control in MEX from a chemical standpoint, in contrast to conventional approaches that rely primarily on physical conditions. This process-driven strategy provides a simple and effective pathway to enhance support removal efficiency and practical applicability in AM. Future studies may include more comprehensive material characterization, such as differential scanning calorimetry, X-ray diffraction curve analysis, Fourier transform infrared spectroscopy, Raman spectroscopy, and X-ray photoelectron spectroscopy of PVA, to provide a deeper understanding of its thermal behavior and molecular structure under different MEX process parameters, including nozzle, chamber, and bed temperature.

## Data Availability

The data presented in this study are available on request from the corresponding author. The data is not publicly available due to ongoing related research.
